# Paradoxical response to disseminated non-tuberculosis mycobacteriosis treatment in a patient receiving tumor necrosis factor-α inhibitor: a case report

**DOI:** 10.1186/1471-2334-14-114

**Published:** 2014-02-28

**Authors:** Takahiro Takazono, Shigeki Nakamura, Yoshifumi Imamura, Taiga Miyazaki, Koichi Izumikawa, Hiroshi Kakeya, Katsunori Yanagihara, Shigeru Kohno

**Affiliations:** 1 Department of Molecular Microbiology and Immunology, Nagasaki University Graduate School of Biomedical Sciences, Nagasaki University School of Medicine, Nagasaki, Japan; 2 Department of Laboratory Medicine, Nagasaki University Hospital, Nagasaki, Japan; 3 Department of Infection Control Science, Graduate School of Medicine, Osaka University, Osaka City, Japan

**Keywords:** Non-tuberculosis mycobacteriosis, *Mycobacterium intracellulare*, Disseminated infection, Relapsing polychondritis, Paradoxical response, Tumor necrosis factor-α inhibitor, Immune reconstitution inflammation syndrome, Non-HIV

## Abstract

**Background:**

Biological agents such as tumor necrosis factor-α inhibitors are known to cause mycobacterium infections. Here, we report a disseminated non-tuberculosis case caused by TNF-α inhibitor therapy and a probable paradoxical response to antimycobacterial therapy.

**Case presentation:**

A 68-year-old man with relapsing polychondritis was refractory to glucocorticoid therapy; adalimumab was therefore administered in combination with oral glucocorticoids. Treatment with 40 mg of adalimumab led to rapid improvement of his clinical manifestations. The administration of tacrolimus (1 mg) was started as the dosage of oral glucocorticoids was tapered. However, the patient developed an intermittent high fever and productive cough 15 months after starting adalimumab treatment. A chest computed tomography scan revealed new granular shadows and multiple nodules in both lung fields with mediastinal lymphadenopathy, and *Mycobacterium intracellulare* was isolated from 2 sputum samples; based on these findings, the patient was diagnosed with non-tuberculosis mycobacteriosis. Tacrolimus treatment was discontinued and oral clarithromycin (800 mg/day), rifampicin (450 mg/day), and ethambutol (750 mg/day) treatment was initiated. However, his condition continued to deteriorate despite 4 months of treatment; moreover, paravertebral and subcutaneous abscesses developed and increased the size of the mediastinal lymphadenopathy. Biopsy of the mediastinal lymphadenopathy and a subcutaneous abscess of the right posterior thigh indicated the presence of Mycobacterium avium complex (MAC), and the diagnosis of disseminated non-tuberculosis mycobacteriosis was confirmed. Despite 9 months of antimycobacterial therapy, the mediastinal lymphadenopathy and paravertebral and subcutaneous abscesses had enlarged and additional subcutaneous abscesses had developed, although microscopic examinations and cultures of sputum and subcutaneous abscess samples yielded negative results. We considered this a paradoxical reaction similar to other reports in tuberculosis patients who had discontinued biological agent treatments, and increased the dose of oral glucocorticoids. The patient’s symptoms gradually improved with this increased dose and his lymph nodes and abscesses began to decrease in size.

**Conclusions:**

Clinicians should consider the possibility of a paradoxical response when the clinical manifestations of non-tuberculosis mycobacteriosis worsen in spite of antimycobacterial therapy or after discontinuation of tumor necrosis factor-α inhibitors. However, additional evidence is needed to verify our findings and to determine the optimal management strategies for such cases.

## Background

Relapsing polychondritis (RP) is a rare systemic disorder characterized by inflammation of the bilateral auricular or tracheal cartilage. Standard treatments for RP include immunosuppressive agents such as corticosteroids, biological agents such as tumor necrosis factor-α (TNF-α) inhibitors and anti-inflammatory agents commonly used to treat rheumatoid arthritis [1]. Patients receiving biological agents have an increased risk for opportunistic mycobacterial and fungal infections [2]. In particular, patients with non-tuberculosis mycobacteriosis (NTM) infections who receive treatments with biological agents are more likely to develop extra-pulmonary or disseminated infections compared with immunocompetent patients [3]. Although biological agents are contraindicated for patients with known NTM, it is difficult to completely eliminate the possibility of infection before starting therapy.

Furthermore, there have been reports of paradoxical responses in patients who developed tuberculosis after discontinuation of anti-TNF-α agents [4,5]. Here, we report a case of disseminated NTM with RP caused by TNF-α inhibitor therapy and a probable paradoxical response to antimycobacterial therapy.

## Case presentation

A 68-year-old man was referred to our hospital for a fever of unknown origin. His medical history was unremarkable except for hypertension. The presence of bilateral auricularis and nasal root inflammation, along with auricular perichondritis that was positive for anti-type II collagen antibodies, led to a diagnosis of RP. Because he was refractory to prednisolone (PSL) glucocorticoid therapy at a dose of 30 mg/day, adalimumab was initiated in combination with oral glucocorticoids after a chest computed tomography (CT) scan indicated that he had no signs of tuberculosis or NTM. Interferon-γ (IFN-γ) release assays (T-SPOT. TB, Oxford Immunotec Ltd., Massachusetts, US) and anti-*Mycobacterium avium* complex (MAC) antibody assays (Capilia MAC, TAUNS laboratories, Inc., Shizuoka, Japan) indicated also negative results. After starting adalimumab treatment (40 mg), his clinical manifestations rapidly improved; therefore, adalimumab was administered 3 times approximately every 2 weeks. The clinical manifestations of RP resolved; moreover, while the PSL dose was gradually tapered to 10 mg/day, treatment with tacrolimus (1 mg/day) was introduced.

**Figure 1:**
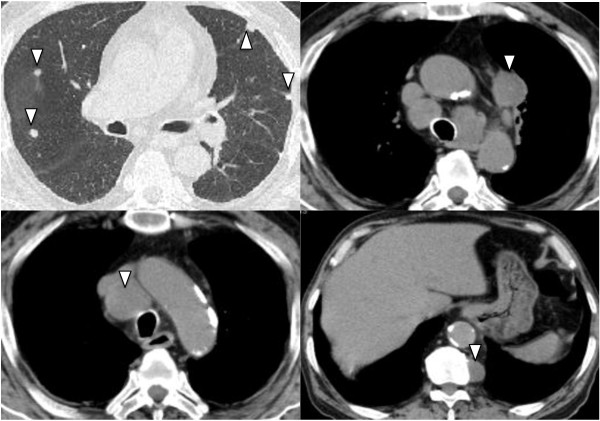
**Computed tomography images of disseminated NTM development.** The white triangles indicate pulmonary nodules, mediastinal lymph nodes, and paravertebral abscess.

The patient subsequently exhibited an intermittent high fever and productive cough 16 months after the RP diagnosis. Laboratory tests showed a normal white blood cell count (8,100/mm^3^) and procalcitonin concentration (0.099 ng/mL), and increased C-reactive protein levels (13.81 mg/dL, normal range < 0.3 mg/dL). The results of all other laboratory tests including liver enzymes, creatinine, and blood urea nitrogen were within normal ranges. A chest CT scan showed granular shadows and multiple nodules in both lung fields with mediastinal lymphadenopathy (Figure 1). *Mycobacterium intracellulare* was isolated from 2 sputum samples; based on these findings, the patient was diagnosed with a pulmonary infection with this NTM. The minimum inhibitory concentrations of the isolated strain for clarithromycin (CAM), rifampicin (RIF), and ethambutol (EMB) were 0.5, 32.0, and 8.0 μg/mL, respectively. Tacrolimus treatment was discontinued. Treatments with CAM, RIF, and EMB at 800, 450, and 750 mg/day, respectively were initiated. The size of the pulmonary nodules and mediastinal lymphadenopathy increased 1 month after the initiation of antimycobacterial therapy. The high fever and general fatigue worsened despite 4 months of treatment; paravertebral and subcutaneous abscesses also developed and the size of the mediastinal lymphadenopathy increased.

**Figure 2:**
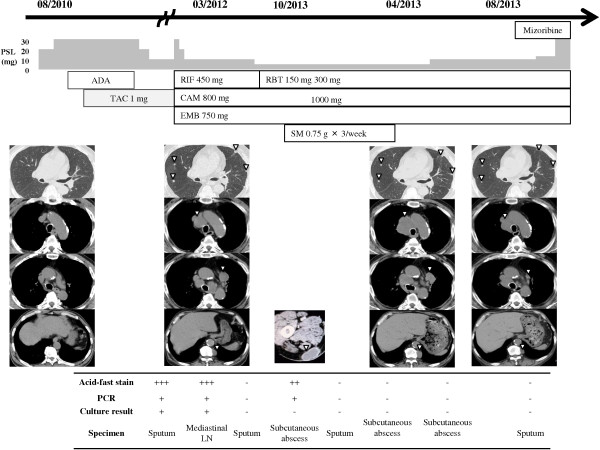
**Clinical course, microbiological test results, treatment regimens, and computed tomography images.** The white triangles indicate pulmonary nodules, mediastinal lymph nodes, paravertebral abscess, and subcutaneous abscess of the right posterior thigh. Abbreviations: ADA, adalimumab; CAM, clarithromycin; EMB, ethambutol; PSL, prednisolone; RBT, rifabutin; RIF, rifampicin; SM, streptomycin; TAC, tacrolimus.

Because biopsy of the mediastinal lymphadenopathy and a subcutaneous abscess of the right posterior thigh indicated infection by *M. intracellulare*, the diagnosis of disseminated MAC infection was confirmed. After 5 months of antimycobacterial therapy with CAM, RIF, and EMB, we changed the medication from RIF to rifabutin at 300 mg/day, increased the dose of CAM from 800 to 1,000 mg/day, and added streptomycin at 0.75 g, 3 times per week; however, the abscesses continued to enlarge. Tests for anti-human T-cell leukemia virus-1 antibodies, anti-human immunodeficiency virus (HIV) antibodies, and antibodies to IFN-γ all yielded negative results. Although we slowly decreased the dose of PSL to 5 mg/day, the mediastinal lymph nodes enlarged even further and abscesses were formed. The mediastinal lymphadenopathy and paravertebral and subcutaneous abscesses also enlarged more and other subcutaneous abscesses developed, although microscopic examination and culture results of sputum and subcutaneous abscess samples indicated negative results 9 months after induction of antimycobacterial therapy. We considered this a paradoxical reaction similar to that noted in previous reports in patients with tuberculosis who discontinued biological agent therapy; therefore, we increased the PSL dosage to 10 mg/day. After this increase, his symptoms gradually improved and the lymph nodes and abscesses began to decrease in size. Due to the deterioration of RP symptoms, the PSL dosage was increased to 30 mg/day and mizoribine was added as a treatment; however, the MAC infection lesions continued to decrease in size. The clinical course, culture results, treatment regimens, and CT scan images are shown serially in Figure 2.

## Discussion and Conclusions

RP itself is not generally considered a risk factor for mycobacterial infections since there have been no reports of RP complicated with NTM. Reports in Thailand and Taiwan found that that most non-HIV patients with disseminated NTM infections exhibited positive results for IFN-γ autoantibodies [6]; however, the patient in the present case exhibited negative results for these antibodies. Patients receiving biological agents are likely to develop extra-pulmonary or disseminated NTM [3], and several fetal cases have been reported [7-9]. Therefore, the disseminated NTM infection in this case was suspected to be related to the use of a TNF α inhibitor.

Mori, et al. reviewed 13 pulmonary NTM cases that had received biological therapy for RA, including infliximab, etanercept, adalimumab, and tocilizumab [10]. The average time from the initiation of biological agent therapy to development of NTM was 10 months (range: 6 weeks to 8.5 years), similar to our case (15 months). Although no cases of disseminated NTM were reported, most cases responded appropriate anti-NTM treatment following discontinuation of biological agents, and none of the cases had a paradoxical reaction after initiation of anti-NTM treatment [10]. Furthermore, in one case, restarting tocilizumab therapy while continuing anti-NTM therapy resulted in a favorable outcome [10,11].

The paradoxical worsening of clinical symptoms and radiologic findings despite effective antimycobacterial therapy may be attributed to immune reconstitution inflammatory syndrome (IRIS). IRIS is defined as the worsening or recurrence of inflammatory symptoms during restoration of an effective immune response against pathogens of opportunistic infections [12,13]. The syndrome has primarily been reported in patients infected with HIV who experience immune reconstitution after initiation of antiretroviral therapy.

It has been suggested that anti-TNF-α agents should be discontinued when *Mycobacterium tuberculosis* infection occurs. However, IRIS has been reported in patients with tuberculosis after discontinuation of anti-TNF-α agents [4,5]. Furthermore, resumption of anti-TNF-α with antimycobacterial drug therapy has been reported to be effective in a tuberculosis case exhibiting a paradoxical response [14]. There are no guidelines or sufficient evidence to recommend a specific course of action in tuberculosis patients administered TNF-α inhibitors who developed a paradoxical response while receiving antimycobacterial treatment.

The reasons for this paradoxical reaction to anti-NTM treatment remain unclear. The combination of adalimumab, tacrolimus, and prednisolone therapies might have resulted in a high mycobacterial load that provoked an immune/inflammatory response upon cessation of adalimumab and tacrolimus treatments.

To our knowledge, this is the first reported non-HIV case of NTM to develop a paradoxical response. Although we considered the presence of a paradoxical response in the present case, the resumption of biological agent treatment was risky because antimycobacterial therapy against NTM is not always effective. Because corticosteroids are an effective treatment for IRIS in patients with HIV, we chose to increase the dose of PSL. Fortunately, our patient responded to this increase, although evidence regarding the optimal dose and duration of steroid therapy remains unclear.

If clinical manifestations of infection worsen despite the administration of antimycobacterial therapy after discontinuation of anti-TNF-α agents, the possibility of a paradoxical response should be considered. Additional evidence is needed to verify our findings and to determine optimal management of such cases.

## Consent

Written informed consent was obtained from the patient for publication of this Case Report and any accompanying images. A copy of the written consent is available for review by the Series Editor of this journal.

## Competing interests

The authors declare that they have no competing interests.

## Authors’ contributions

TT, SN, YI, TM, and HK were responsible for clinical testing and treatments. KI and SK supervised the work and helped to draft the manuscript. KY participated in the microbiological work-ups. All authors have read the manuscript and accepted the final version.

## Pre-publication history

The pre-publication history for this paper can be accessed here:

http://www.biomedcentral.com/1471-2334/14/114/prepub
